# Immunotherapy in the Treatment of Urothelial Bladder Cancer: Insights From Single-Cell Analysis

**DOI:** 10.3389/fonc.2021.696716

**Published:** 2021-05-26

**Authors:** Jingyu Zang, Kaiyan Ye, Yang Fei, Ruiyun Zhang, Haige Chen, Guanglei Zhuang

**Affiliations:** ^1^ State Key Laboratory of Oncogenes and Related Genes, Shanghai Cancer Institute, Ren Ji Hospital, School of Medicine, Shanghai Jiao Tong University, Shanghai, China; ^2^ Department of Urology, Ren Ji Hospital, School of Medicine, Shanghai Jiao Tong University, Shanghai, China

**Keywords:** urothelial bladder cancer, immunotherapy, immune checkpoints, single-cell analysis, tumor microenvironment

## Abstract

Urothelial bladder cancer (UBC) is a global challenge of public health with limited therapeutic options. Although the emergence of cancer immunotherapy, most notably immune checkpoint inhibitors, represents a major breakthrough in the past decade, many patients still suffer from unsatisfactory clinical outcome. A thorough understanding of the fundamental cellular and molecular mechanisms responsible for antitumor immunity may lead to optimized treatment guidelines and new immunotherapeutic strategies. With technological developments and protocol refinements, single-cell approaches have become powerful tools that provide unprecedented insights into the kaleidoscopic tumor microenvironment and intricate cell-cell communications. In this review, we summarize recent applications of single-cell analysis in characterizing the UBC multicellular ecosystem, and discuss how to leverage the high-resolution information for more effective immune-based therapies.

## Introduction

Urothelial bladder cancer (UBC) accounts for more than half a million new diagnoses and 212,536 deaths annually (1). Approximately 75% of primary UBC cases are non-muscle invasive bladder cancer (NMIBC), which is typically treated with transurethral resection (TURBT) followed by intravesical instillation of chemotherapeutics or Bacillus Calmette-Guérin (BCG) (2–4). Muscle invasive bladder cancer (MIBC) is the minor yet more lethal disease modality, for which optimizing medical care and reducing morbidity after radical cystectomy are major goals (4–6). Clinical management of UBC patients is undergoing rapid changes as tumor immunotherapies, molecular targeted agents, and antibody-drug conjugates have increasingly become viable options (7, 8). In particular, immune checkpoint inhibitors (ICIs) harness patients’ own immune system to counteract malignant cells and represent a major breakthrough in recent years. Since 2016, up to five different ICIs targeting programmed cell death protein 1 (PD-1), i.e., pembrolizumab and nivolumab, or programmed cell death ligand 1 (PD-L1), i.e., atezolizumab, avelumab and durvalumab, are approved by FDA for the treatment of late-stage urothelial carcinoma. However, only about 20% of UBC patients show an effective response to anti-PD-1/PD-L1 monotherapy, which often fails to translate into long-term survival benefit compared with standard chemotherapy (9–14).

Extensive studies have been focused on dissecting the cellular and molecular mechanisms underlying the immune response of UBC, in order to identify clinical biomarkers to predict ICI treatment efficacy, and to design novel single or combination trials of more effective regimens (15–17). Accumulative evidence suggests that tumor cells and the associated nontumor constituents in UBC microenvironment interact to modulate cancer immunogenicity and immunotherapeutic outcomes (18–20). Therefore, a comprehensive characterization of diverse cell types and states in the context of UBC oncogenesis and treatment is of paramount importance. Conventional methodologies often yield incomplete and mixed signals attributable to both malignant and nonmalignant cells, precluding precise evaluation on the biological determinants of ICI effects (21, 22). The emerging single-cell technologies, along with blossoming bioinformatic tools, promise to provide a high-resolution tumor immune landscape and exert a prominent impact on the field of UBC immunotherapy. By analyzing the genomic (23), transcriptomic (24–27), and proteomic (28, 29) features at a high-throughput manner, single-cell approaches generate new insights into complex systems like UBC. The rich information allows to infer heterogeneous cellular compositions, study dynamic cell state transitions, and construct cell-cell communication networks, which collectively may transform our understanding of responsiveness and resistance to PD-1/PD-L1 inhibitors, and fuel rational development of new immune-modulating therapies and combinations.

In this review, we update the current progress of cancer immunotherapy in UBC, summarize the applications of cutting-edge single-cell analysis in decoding the tumor multicellular ecosystem, and discuss future prospects for using these high-dimensional multi-faceted data to guide more effective immune checkpoint therapies.

## The Advances and Challenges of Immunotherapy for UBC

### Conventional Therapies for UBC

**Figure 1:**
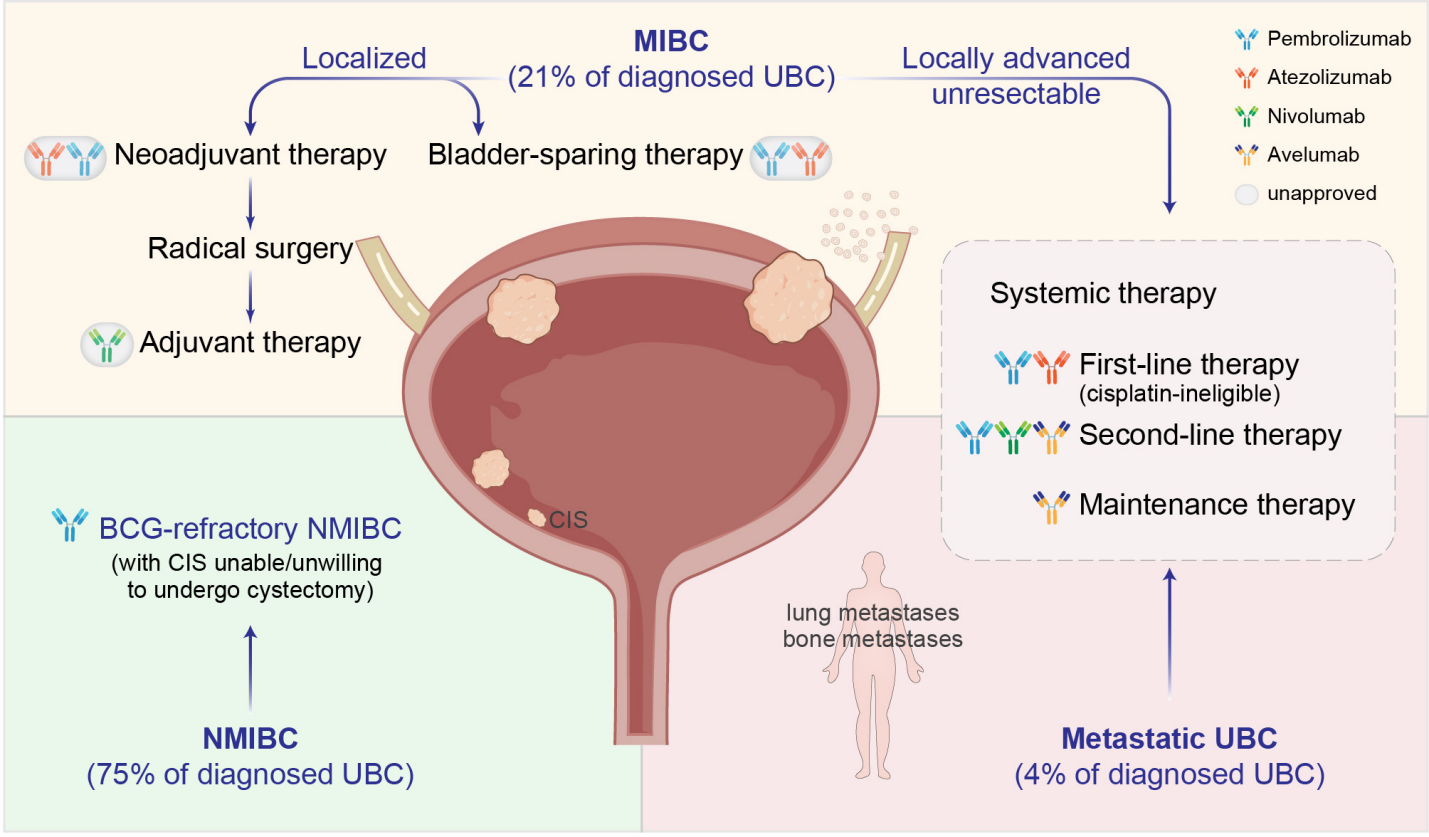
Clinical management of UBC with immune checkpoint inhibitors (ICIs). Dark-colored antibodies: currently approved ICIs; circled light-colored antibodies: in clinical trials. BCG, Bacillus Calmette-Guérin; CIS, carcinoma *in situ*.

UBC can be divided into NMIBC and MIBC according to the depth of tumor invasion. The two disease entities have unique pathological characteristics and distinct standard treatment guidelines (7). NMIBCs refer to neoplasms staged as Ta, T1, or CIS (carcinoma *in situ*), and are usually managed with TURBT followed by a single dose of intravesical chemotherapy to kill free-floating tumor cells. After the initial TURBT, patients with intermediate or high likelihood of recurrence will receive adjuvant intravesical BCG as maintenance therapy to reduce the risk of progression (30, 31). For patients who have intolerable adverse effects or fail BCG therapy owing to persistent or worsening disease, the most effective treatment is radical cystectomy (32). UBC lesions invading the muscular layer or perivesical tissues (T2-T4) are categorized as MIBC. Neoadjuvant platinum-based chemotherapy (NAC) plus radical cystectomy is the standard of care for localized MIBC. However, only 20% of patients are eligible to receive NAC (33), and almost half of them still have residual disease after NAC, leading to poor prognosis (34). Moreover, approximately 4% of newly diagnosed UBCs present distal metastasis (4), for which the mainstay of treatment has long been systemic cytotoxic chemotherapy. It is noteworthy that bladder preservation is associated with better quality of life and therefore under active investigations as an attractive alternative in the management of both NMIBC and MIBC. While the survival improvement achieved with conventional therapies has reached a plateau and there are few advances in UBC treatment over the past decades, the paradigm is being considerably shifted with the development and application of immune checkpoint therapeutics (**Figure 1**).

### Immune Checkpoint Inhibitors for UBC

#### Second-Line Therapy

Second-line ICIs are suitable for UBC patients with advanced disease who have previously received platinum-based chemotherapy and subsequently progressed or metastasized. In the KEYNOTE-045 phase III trial (11), patients receiving pembrolizumab experienced improved overall survival (OS) compared to second-line physician’s choice of chemotherapy (10.3 *vs* 7.4 months; HR, 0.73 [95% CI, 0.59-0.91]; *P* = .002). Based on these results, pembrolizumab was approved as a second-line treatment for those whose disease progressed during or after platinum-based chemotherapy. In addition, avelumab (JAVELIN Solid Tumor) (12) and nivolumab (CheckMate 275) (13) also gained accelerated FDA approval as second-line agents, both of which demonstrated clinical benefit in the advanced or metastatic setting.

Unfortunately, a major setback emerged as some ICIs originally granted accelerated approval on the basis of phase II trials did not achieve clinical confirmation in subsequent phase III studies. For example, despite promising phase II data (IMvigor210) (14), atezolizumab did not improve OS in a phase III randomized trial (IMvigor211) compared with second-line chemotherapy (11.1 *vs* 10.6 months; HR, 0.87 [95% CI, 0.63-1.21]; *P* = .41) (35). Likewise, according to the phase III study (DANUBE), durvalumab failed to prolong OS (14.4 *vs* 12.1 months; HR, 0.89 [95% CI, 0.71-1.11]; *P* = .30) (36). As a result, these two drugs have been officially withdrawn from the second-line treatment of bladder cancer (7).

#### First-Line Therapy

Pembrolizumab and atezolizumab were given accelerated approval for the first-line treatment of cisplatin-ineligible advanced or metastatic UBC, following KEYNOTE-052 (37) and IMvigor210 (14) phase II trials. Nevertheless, treatment with pembrolizumab and atezolizumab only yielded an objective response rate (ORR) of 24% and 23%, respectively. Both studies assessed the ICI efficacy in relation to PD-L1 expression status and found that PD-L1 score alone was not sufficient to precisely predict the treatment responsiveness. Other potential predictive biomarkers, such as tumor mutational burden (TMB) and relevant gene expression profiling (GEP), are being investigated without consensus guidelines in practice.

In contrast, three recent trials with cisplatin-eligible patients consistently showed that first-line ICI monotherapy was not superior to chemotherapy in unresectable locally advanced or metastatic UBC. All these large randomized phase III trials, i.e., IMvigor130, KEYNOTE-361, and DANUBE, observed similar performance of three ICI drugs and platinum-containing chemotherapy in the front-line setting (36, 38–40). Even though the chemoimmunotherapy combo showed some efficacy signals, this result, as it currently stands, appears not to be practice-changing. The next step is to further explore the combination of different ICIs, as well as immunotherapy plus other targeted drugs, in multiple ongoing phase III trials including CheckMate 901, NILE, LEAP-011 and EV-302 (41–44).

#### Maintenance Therapy

Javelin Bladder 100 was the first phase III trial to establish the role of maintenance immunotherapy immediately following first-line chemotherapy in advanced or metastatic urothelial carcinoma (45). For patients who did not have disease progression with standard chemotherapy (4-6 cycles of gemcitabine plus cisplatin or carboplatin), the addition of maintenance avelumab to best supportive care significantly prolonged overall survival (21.4 *vs* 14.3 months; HR, 0.69 [95% CI, 0.56-0.86]; *P* = .001). The evident improvement of patient outcomes has led to the FDA approval of avelumab as maintenance therapy in this disease setting (46). However, although no new safety signals were identified, there was a higher incidence of adverse events in the avelumab group than in the control group and 11.9% of the patients receiving maintenance avelumab discontinued the therapy because of side effects.

#### Adjuvant Therapy

The role of adjuvant immunotherapy in MIBC patients after cystectomy remains to be elucidated by prospective clinical studies. One phase III trial (IMvigor010) did not meet its primary endpoint of improved disease-free survival in the atezolizumab group over observation (19.4 *vs* 16.6 months; HR, 0.89 [95% CI, 0.74-1.08]; *P* = .24) (47). On the other hand, first results from the phase III CheckMate 274 trial supported use of nivolumab in MIBC after radical surgery (48). Additional high-quality evidence is required to formulate treatment guidelines recommending adjuvant ICIs for MIBC patients with high-risk pathologic features.

#### Neoadjuvant Therapy

Clinical trials of perioperative immunotherapy are ongoing in patients with advanced urothelial carcinoma. In PURE-01 phase II study, 42% of patients treated with pembrolizumab achieved pathologic complete response (pCR) and up to 54% downstaged to pT1 or lower disease (49). The ABACUS phase II study reported a pCR rate of 31% and the majority of patients underwent surgery successfully after neoadjuvant atezolizumab therapy (50). Encouraged by these results, a series of phase III trials assessing ICIs as monotherapy or in combination have been initiated (51). Although neoadjuvant ICIs demonstrate promising antitumor activity, they also pose new challenges in clinical decision-making (52). First, the evaluation criteria of neoadjuvant therapy efficacy are not unified at present. Second, when the patients meet the standard of surgical treatment, and whether curative surgery should be averted or delayed if pCR is achieved are all issues to be considered (4). Third, not all patients benefit from neoadjuvant ICI treatment and selective biomarkers are urgently needed. Finally, during the treatment, immune cells may infiltrate into tumor tissues, causing lesion enlargement and pseudoprogressive imaging findings. Therefore, distinguishing between real progression and so-called “tumor flare” is of necessity (53).

#### Bladder-Sparing Therapy

As a reasonable alternative to radical cystectomy, trimodal therapy (TMT) combines maximal TURBT with concomitant radiosensitizing chemotherapy and external-beam radiotherapy to devise bladder-sparing strategies in well-selected patients. Given that ICIs may further augment the immune response triggered by radiotherapy-induced tumor cell death (5), several studies are evaluating the potential synergy between chemoradiation and immunotherapy, including KEYNOTE-992 and SWOG S1806, two phase III randomized trials investigating ICIs in bladder-sparing treatment of MIBC (4, 54, 55). Of particular note, the incorporation of clinical biomarkers is a major consideration to carefully gauge which patients are optimal candidates for organ-preserving opportunities and if salvage cystectomy is needed during the course of less aggressive treatment.

#### BCG-Refractory NMIBC

For patients having BCG-refractory NMIBC with CIS who are unable or unwilling to undergo cystectomy, pembrolizumab was recently approved on the basis of results from KEYNOTE-057 phase II study (56, 57). The complete response (CR) rate was 40.6%, and nearly half of responding patients experienced a CR lasting at least 12 months. During the course of pembrolizumab treatment, no patient’s disease progressed to muscle-invasive or metastatic bladder cancer. Additional trials evaluating the use of immunotherapy in NMIBC including the phase III KEYNOTE-676 are underway (58).

### Mechanism of Action for PD-1/PD-L1 Checkpoint Blockade

**Figure 2:**
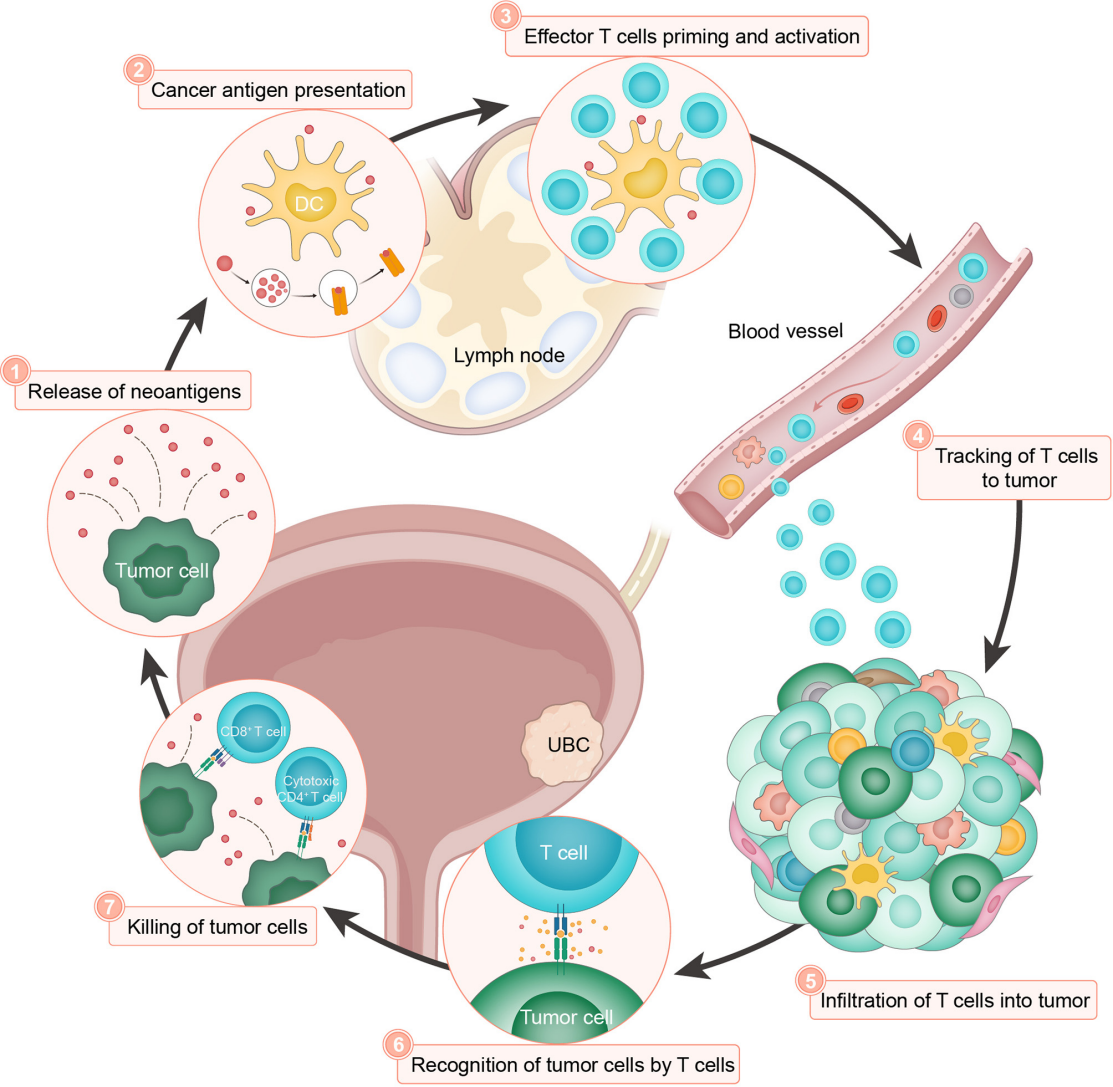
The cancer-immunity cycle in UBC. The cancer-immunity cycle is based on the illustration by Chen and Mellman (62). The cancer-immunity cycle can be divided into seven major steps, starting with the release of neoantigens from the cancer cells (step 1) and ending with the killing of cancer cells (step 7). DC, dendritic cell.

To fulfill a robust and durable clinical benefit of tumor immunotherapy, immense efforts have been taken to comprehensively understand the mechanism of action for PD-1/PD-L1 checkpoint blockade (20). Under physiological conditions, to avoid damaging autologous cells during prolonged immune response, the activation of T lymphocytes is strictly counterbalanced by inhibitory signals, such as immune checkpoint pathways, resulting in hyporesponsive adaptation while limiting detrimental immunopathology. As a particularly important regulatory axis, PD-L1 binds to the PD-1 receptor and functions as the brake of immune cells by suppressing lymphocyte proliferation and cytokine secretion (59, 60). In the process of neoplastic initiation and development, accumulating somatic aberrations give rise to tumor-specific neoantigens, which can be recognized by host defense system as nonself (60, 61). To elicit effective immune responses, a serial of stepwise events, termed the ‘cancer-immunity cycle’ (**Figure 2**), must proceed and expand iteratively (62). In brief, the release of neoantigens (step 1) and their presentation by dendritic cells (step 2), is followed by effector T cell priming and activation (step 3), trafficking to (step 4) and infiltrating the tumor bed (step 5), consequently resulting in recognition (step 6) and killing of target cells (step 7) to deliver additional tumor-associated antigens (step 1 again). This cyclic process leads to an accumulation of immune-stimulatory factors that amplify and broaden T cell responses. However, the generation of immunity to cancer is not always optimal, and can be halted by immune regulatory feedback mechanisms. For example, tumor cells often abnormally express PD-L1 to engage PD-1 and resist immune attack. Currently approved ICIs in UBC target the PD-1/PD-L1 interaction and reinvigorate the cytotoxic capacity of T lymphocytes against malignant cells (63). Nonetheless, other modes of immunosuppression may exist to impair the intact cancer-immunity cycle and tumor responsiveness to ICI treatment (20, 64–66). At present, immunohistochemistry staining, lymphocyte cell surface protein labeling, and bulk-level high-throughput sequencing, are commonly used to analyze the relevant immune characteristics. However, these approaches yield incomplete or mixed signals from the multicellular microenvironment, which largely ignore biological complexity and intratumoral heterogeneity. With recent advances in single-cell technologies, comprehensive profiling of tumor immune components and their functional properties would facilitate the characterization of diverse cell types and states, shed light on the inherent immune biology related to bladder cancer, and provide unique and nuanced insights into primary or acquired resistance to anticancer immunotherapies (67).

## Applying Single-Cell Technologies to UBC

### Samples for Consideration

Generally, FFPE (formalin-fixed and paraffin-embedded) or snap-frozen clinical samples, though readily available, can only be used for single-nucleus sequencing (68). The method may work well for DNA but not RNA detection, because the profiling of nuclear RNA ignores its cytoplasmic counterpart and cannot represent the whole picture of cellular transcriptome (69). Therefore, single-cell workflows based on viable cell suspension remain the preferred approach, despite technical challenges associated with immediate collection and processing of fresh tissues (68). In addition, longitudinal observation of cell types and states during a treatment course is of vast importance but requires repeated tumor biopsies, which is usually unfeasible due to ethical issues. To circumvent this limitation, murine bladder cancer induced by continuous exposure to carcinogenic chemicals serves as an alternative system. Genetically engineered mouse model (GEMM) or patient-derived xenograft (PDX) in immunodeficient animals can also be exploited (18, 70, 71). Of note, co-engraftment of human hematopoietic stem cells partly recapitulates the human tumor immune microenvironment and may be helpful to enable interactions between PDX and immune cells, allowing for experimental evaluation of immunotherapy (71). Moreover, patient-derived organoid (PDO) provides an ex vivo platform for studying tumor evolution and drug response (72, 73). Of special note, the urine from UBC patients, compared to peripheral blood, is a faithful and rich source of tumor-derived materials including DNA, protein, and exfoliated cells (74–77). Thus, single-cell analysis of urinary lymphocytes can be potentially employed as a noninvasive strategy to monitor tumor immune microenvironment at the cellular level. Indeed, there is evidence that the number of urinary lymphocytes is significantly increased following intravesical BCG instillation in patients with NMIBC (78). In MIBC, urine-derived and tumor-infiltrating lymphocytes closely resemble each other in immune checkpoint landscape and T cell receptor repertoire (75). Therefore, urinary exfoliated immune cells represent a dynamic liquid biopsy for UBC that may be subjected to single-cell interrogation.

### Single-Cell Analysis of UBC

#### Single-Cell RNA Sequencing

To date, single-cell RNA sequencing (scRNA-seq) is the most mature single-cell genomic approach and has a wide spectrum of novel analytic tools to facilitate data interpretation (79–83). The major application of scRNA-seq is to systematically characterize heterogeneous cell types and molecular states in both healthy tissues and malignant conditions. For instance, a recent study created a single-cell transcriptomic map of human and mouse bladders, unveiling both conservative and heterogeneous aspects of bladder evolution (24). A subsequent study generated a single-cell atlas of primary bladder carcinoma and uncovered the protumor function of inflammatory cancer-associated fibroblasts (25). Sfakianos et al. identified lineage plasticity of human and mouse bladder cancer at single-cell resolution, which may contribute to innate tumor heterogeneity (26). In addition, comparative scRNA-seq analysis between pre- and post-tipifarnib MIBC PDX revealed an increased population of dormant drug-refractory tumor cells and simultaneous remodeling of tumor-supporting microenvironment (27).

#### Single-Cell T Cell Receptor Sequencing

T cells play a vital role in adaptive immunity and represent the major target of antitumor immunotherapy (84). T cell receptor (TCR) locates on the surface of T cells and recognizes antigenic peptides presented by major histocompatibility complex (MHC) molecules. Genetic recombination creates a diverse TCR repertoire during ontogeny or disease. The majority of TCRs are comprised of α and β chains (85), which can be reconstructed by single-cell T cell receptor sequencing (scTCR-seq) to elucidate T cell clones involved in immune response (86). Furthermore, the combined analysis of scRNA-seq and paired scTCR-seq may link the cellular phenotypes with specific clonotypes of T lymphocytes. Using this approach, Oh et al. demonstrated that CD4^+^ T cells in bladder cancer exhibit multiple distinct tumor-specific states of regulatory T cells and cytotoxic CD4^+^ T cells, which were clonally expanded (87, 88). In contrast, the states and repertoires of CD8^+^ T cells, which were traditionally recognized as the main killers in immuno-oncology (89), were indistinguishable in bladder tumors compared with non-malignant tissues.

#### Single-Cell DNA Sequencing

According to the genomic coverage, single-cell DNA sequencing (scDNA-seq) mainly includes whole-genome scDNA-seq, whole-exome scDNA-seq, and panel scDNA-seq detecting a few genes of interest. Whole-genome or whole-exome scDNA-seq covers large genomic regions but is limited by sequencing depth, while panel scDNA-seq focuses on a narrow list of target genes but can achieve higher throughput and sequencing depth (90). Despite in its infancy, scDNA-seq has been applied to identify driver mutations and investigate cancer evolution. A notable example was that Yang et al. demonstrated the co-mutation of *ARID1A*, *GPRC5A*, and *MLL2* were the major self-renewal driver of human bladder cancer stem cells. Through phylogenetic analysis, the study also suggested the biclonal origin of bladder cancer stem cells from both bladder cancer non-stem cells and bladder epithelial stem cells (23).

#### CyTOF Mass Cytometry

Cytometry by time of flight (CyTOF) adopts the single-cell format of flow cytometry technique for multiparameter detection of protein expression using the precision of mass spectrometry (91). By employing a pre-selected panel of metal-labeled antibodies, dozens of surface or intracellular markers can be quantified at the same time to infer the potential identity and functionality of target cells. In a study to evaluate NMIBC response to BCG treatment, CyTOF was employed to observe a decreasing trend of T cell subsets in peripheral blood and corresponding tissue recruitment of immune cells in treated tumors (28), thus supporting the rationale of combining immunotherapy to overcome BCG resistance in NMIBC patients. Likewise, Megan et al., *via* CyTOF and RNA-seq analyses, uncovered higher CD8^+^ T cell populations in murine bladder cancer upon DDR2 depletion and anti-PD-1 treatment, implying that DDR2 inhibition might fuel tumor response to ICIs (29).

### Emerging Single-Cell Technologies

As an evolving field, numerous novel single-cell technologies are in rapid development to extract additional layers of biological information. For example, surface protein levels can also be measured in single cells by oligonucleotide-barcoded antibodies, as illustrated by various methods including CITE-seq and REAP-seq (92, 93). Another relevant knowledge tier is the cellular epigenetic state, and recently described scATAC-seq and scDNase-seq, among others, enable high-throughput examination of chromatin accessibility at single-cell resolution (94). One key attribute of tumor ecosystem is the spatial distribution of cellular niches which directly determines physical cell-cell interactions and intercellular signaling communications (95). Specialized tools integrating spatially resolved transcriptomics and advanced imaging infrastructure characterize gene expression profiles within a broader tissue context. Additionally, single-cell metabolomics is being added to the toolbox for metabolic deconvolution but currently is too premature to allow large-scale applications (96). We envision that future studies in UBC leveraging these rising single-cell technologies hold a great deal of promise to enrich our understanding of disease biology and accelerate the discovery of new therapeutic strategies.

## Potential Insights From Single-Cell Analysis

### Tumor Multicellular Ecosystem

It is increasingly evident that various cell populations residing at neoplastic lesions and the interplay of these cellular compartments strongly affect cancer progression and response to immunotherapeutics. Recently, single-cell studies have provided in-depth insights into the composition and architecture of tumor multicellular ecosystem in UBC. By profiling the transcriptome of 52,721 single cells from bladder urothelial carcinoma or peritumor mucosa samples, Chen et al. discovered seven annotated cell types including epithelial cells, endothelial cells, fibroblasts, B cells, myeloid cells, T cells, and mast cells (25). Despite the presence of adaptive lymphocytes, cancer cells exhibited intrinsic ability to evade immune surveillance by expressing lower levels of MHC-II molecules than normal epithelial cells. In addition to diverse clusters of myeloid cells, two distinct fibroblast subtypes were identified in UBC: inflammatory fibroblasts and myofibroblasts with the former expressing various cytokines and displaying pro-proliferative effects. It is especially noteworthy that a number of important observations in other cancers are recapitulated in UBC. First, unrelated human malignancies surprisingly harbour analogous cell types (97). Second, tumor cells consistently show a patient-specific expression pattern, whereas immune and stromal infiltrates are more homogenous across different subjects (98–101). Third, both innate and adaptive immunity are involved in cancer pathogenesis (102, 103). Fourth, individual cellular components crosstalk with each other and form intricate interaction networks (104). Collectively, the single-cell transcriptomic atlas reveals cellular and molecular complexity of the UBC ecosystem, and highlights ongoing intratumoral immune suppression as a potential therapeutically actionable abnormality.

### T Cell Subsets and States in Cancer

The T cell infiltrates in human cancer largely determine natural disease behavior and also the probability of immunotherapeutic response. It has long been known that intratumoral T lymphocytes span across a spectrum of subsets and states, with the simplest distinction of CD4^+^ and CD8^+^ T cell populations (84). While the evidence for a predominant function of CD8^+^ T cells in tumor control is compelling, the role of CD4^+^ T cells used to be conceptualized as indirect by either supporting CD8^+^ T cell-mediated tumor killing *via* a helper phenotype or restricting such processes *via* a regulatory phenotype (105). Oh et al. applied scRNA-seq and paired scTCR-seq to characterize the immune milieu of 7 MIBC patients (87). Reminiscent of heterogeneous T cell infiltrates defined in previous studies (106–108), a diverse range of T cell subtypes also existed in UBC, including both CD4^+^ and CD8^+^ T cells that could be further clustered into different functional subgroups. However, in contrast to the canonical view, two cytotoxic CD4^+^ T cell populations were unexpectedly identified in bladder cancer that correlated with a significantly increased likelihood of clinical response to PD-L1 inhibition (87). Importantly, cytotoxic CD4^+^ T Cells were clonally expanded in tumor lesions and possessed lytic capacity against autologous tumor cells in an MHC class II-dependent fashion. Although there are a number of caveats about this elegant work, e.g., mixed analysis of both treatment-naive and chemotherapy or immunotherapy-treated samples, the findings have substantial implications by pinpointing the underappreciated potential of cytotoxic CD4^+^ T cells in UBC. Considering that ICIs ultimately rely on the activity of a pre-existing or newly induced tissue-resident T cell pool to achieve tumor elimination, the identification of cytotoxic CD4^+^ T cells therefore redefines our thinking regarding UBC immunotherapies and further raises several crucial questions, such as whether these cells are associated with an ongoing tumor-specific immune response and how the current checkpoint inhibitors would impact them.

### Tumor Cell Heterogeneity and Plasticity

As aforementioned, single-cell analyses highlight the divergent nature of cancer cells underlying the prevalent heterogeneity between and within individual tumors. This observation is perhaps not surprising given that each malignant cell is featured by a unique evolutionary trajectory and inherent biological stochasticity (109–111). Despite the diversity, specific transcriptional states may still be shared across a subpopulation of neoplastic cells or cancer patients. In the case of UBC, a string of studies on bulk gene expression profiles have identified distinct molecular subtypes in MIBC, including luminal-papillary, luminal, basal-squamous, luminal-infiltrated, and neuronal (16). Such a classification again attests to the differential transcriptome-wide programs operating in separate tumor cells and can be useful to stratify patients for prognosis or treatment. Remarkably, several reports suggest that responses to chemotherapy and immunotherapy are enriched in certain MIBC subtypes (112). Recent scRNA-seq of human and murine bladder cancers, however, revealed a hidden layer of complexity by demonstrating marked cell-autonomous heterogeneity and multidirectional plasticity of the urothelial lineage (26). Therefore, although the initial predominant molecular subtypes may substantially dictate UBC progression kinetics and therapeutic response, they also undergo dynamic changes during tumor growth or clinical treatment, e.g., chemotherapy (113) and immunotherapy. In turn, this subtype transition will presumably engender functional consequences, which should be discreetly considered in the use of immune-modulating agents.

## Outstanding Questions and Future Prospects

Novel applications of single-cell technologies in characterizing UBC are currently limited in comparison to the rapid progress that has been seen in other human malignancies (114–117). As a result, our understanding of bladder cancer cell hierarchy and tumor microenvironment is not complete, and more studies will be required to better delineate the abundance, localization, and functional orientation of each cellular component. For instance, the innate immune landscape like myeloid cell populations in UBC remains to be fully elucidated by single-cell analysis. Likewise, the makeup of antigen presenting cells as a crucial factor for efficient immune activation has been insufficiently described. Ideally, all the information should be decoded in a spatiotemporal context (19). As a relevant example, tertiary lymphoid structures (TLS) in human cancer, which are highly organized cellular aggregates resembling lymph nodes, have recently emerged as key sites for the generation of antitumor immunity with a prominent impact on disease outcome and immunotherapeutic response (118–121). We anticipate that single-cell analysis will soon become essential to resolve TLS composition, location, density and degree of maturation during UBC tumorigenesis and treatment.

The success of cancer immunotherapy has prompted intensified interest in defining the specific effector immune cells and fundamental mechanisms responsible for anti-tumor immunity. In addition, certain oncogenic pathways and transcriptional programs in malignant cells are associated with intrinsic sensitivity or resistance to immunotherapeutics (122, 123). These cumulative findings hold enormous promise to facilitate biomarker identification that can predict or monitor which patients would benefit from immunotherapy. The treatment stratification and surveillance are of paramount importance for UBC as ICI therapy is being aggressively advanced into the neoadjuvant and bladder-sparing settings, where inappropriate regimens could be potentially detrimental. Unfortunately, individual parameters have been proved unreliable and such a model has to take different elements that affect tumor-host interactions into account (17, 124). Thus, taking advantage of cutting-edge approaches such as single-cell sequencing and mass cytometry, which enable high-dimensional molecular analyses during the whole course of ICI treatment, will be valuable to simultaneously probe a wide range of immune subsets and regulators, and systemically nominate biomarker candidates for further detailed investigations.

Beyond anti-PD-1/PD-L1 monotherapy, a breadth of basic research and clinical trials are ongoing to explore the strategy of combined therapy in UBC treatment, such as different ICI pairs (e.g., nivolumab and ipilimumab), immunotherapy and targeted small molecules (e.g., erdafitinib) or antibody-drug conjugates (e.g., enfortumab vedotin and sacituzumab govitecan) (125–127). At the moment, many drug-development pipelines evaluate the efficacy of combo agents on the basis of a simple try-and-see approach. There are continued concerns about whether adverse effects will be additive and whether the antitumor response will be improved. We argue that data-driven design of synergistic drug combinations may most likely make a breakthrough for maximizing patient benefit from these transformative therapies, based on a comprehensive understanding of the bladder cancer ecosystem at the single-cell level.

Ultimately, the multiparametric data derived from single-cell technologies ought to assist UBC patient care and inform treatment recommendations. Achieving the ambitious goal will need joint efforts to develop standard operating procedures for benchmarking and implementing single-cell workflows that meet ethical, regulatory, and temporal requirements. With all foreseeable challenges, this venture would be imperative to transform bladder cancer management and necessitate very close collaboration among physicians, basic researchers and translational scientists. Recently launched large-scale initiatives, including the Human Tumor Atlas Network (HTAN) and the Tumor Profiler (TuPro) study, are poised to accelerate the standardization of key protocols, best-practice guidelines, quality control solutions, metadata schemata, and analytic pipelines (128, 129). These projects may lead to refined diagnostics in precision oncology and pave the way for the translation of single-cell profiling into clinical decision-making.

## Conclusions

**Figure 3:**
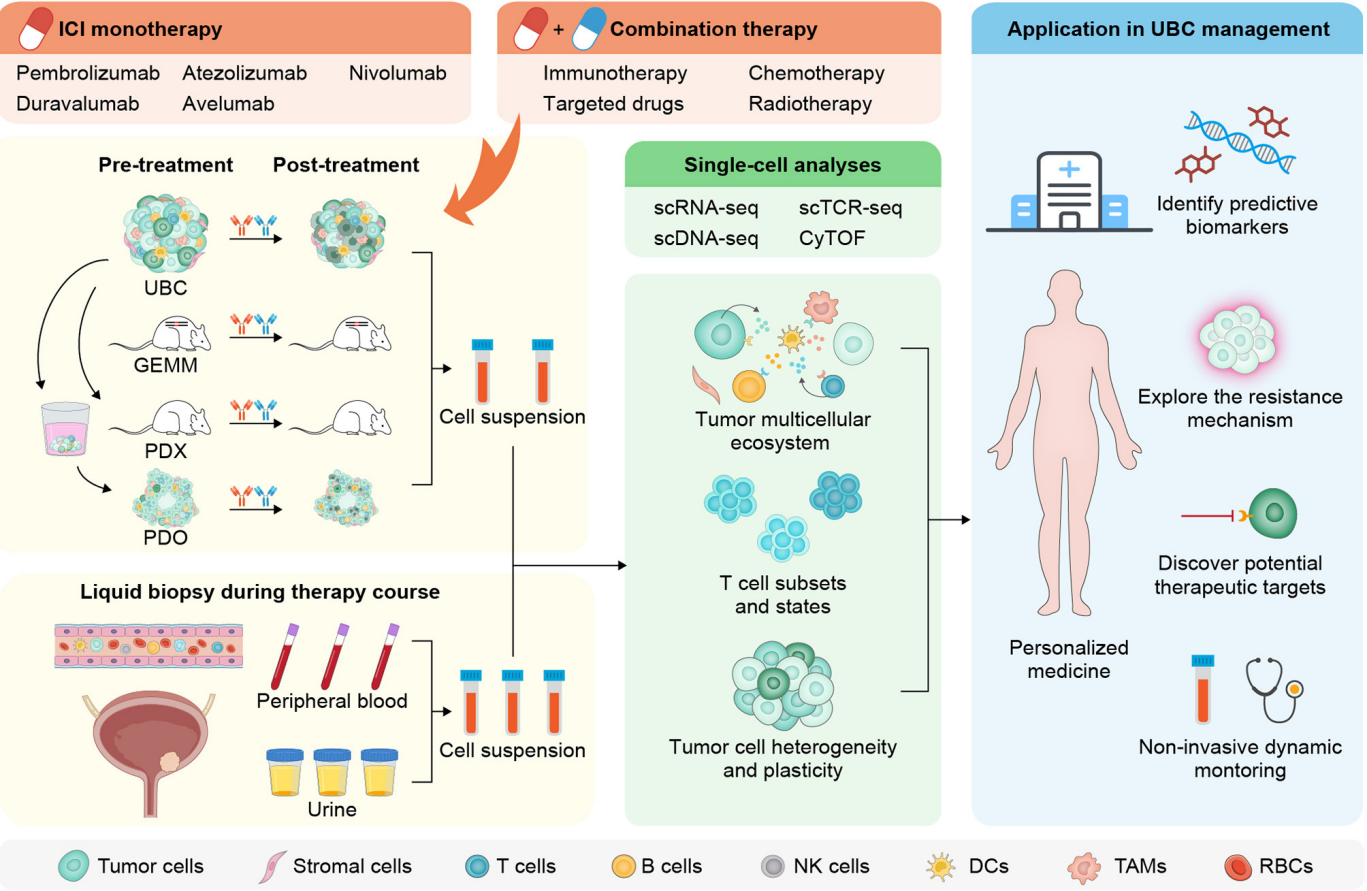
Workflow and applications for single-cell analysis in the immunotherapy of UBC. After sampling before and after ICI monotherapy or combination therapy from UBC patients or alternative experimental models, single-cell suspensions with myriad cell types and states are preprocessed for downstream analysis. The longitudinal and noninvasive single-cell profiling on liquid biopsies from peripheral circulation or urine may aid dynamic monitoring of UBC patients (left panel). A variety of single-cell technologies enable comprehensive assessment of tumor, immune, and stromal cells to yield high-dimensional information (middle panel). Findings from the single-cell approaches promise to allow a detailed dissection of the mechanisms underlying immunotherapeutic response and resistance, and facilitate designing rational single or combination immune-based therapies (right panel). GEMM, genetically engineered mouse model; PDX, patient-derived xenograft; PDO, patient-derived organoid; scRNA-seq, single-cell RNA sequencing; scTCR-seq, single-cell T cell receptor sequencing; scDNA-seq, single-cell DNA sequencing; CyTOF, cytometry by time of flight; NK, natural killer; TAMs, tumor-associated macrophages; RBCs, red blood cells.

The recent decade has witnessed unprecedented advances in the clinical management of urothelial carcinoma with the advent of various ICIs. The ever-expanding applicable range of ICI therapies in UBC highlights the significant potential of immune-targeted agents and advocates a more thorough interrogation of their mechanistic underpinnings. Despite remaining questions, a number of studies using high-resolution single-cell techniques begin to reveal the identity and state of multiple cell types, the variety and uniqueness of tumor-infiltrating T lymphocytes, as well as the heterogeneity and plasticity of bladder cancer cells. This wealth of information has allowed a better understanding of dysfunctional antitumor immunity in UBC and variable responses to immunotherapy across patients (**Figure 3**). However, single-cell methods are still nascent, and over the coming years, an emerging repertoire of multiplexed assays with spatial readout will further enhance their capabilities. In addition, single-cell approaches coupled with noninvasive blood- or urine-based liquid biopsies are instrumental to dynamically evaluate therapeutic efficacy and monitor disease relapse. With these innovative toolkits available, future work should focus on establishing a molecular taxonomy for each cell composition, defining the cellular geography within neoplastic lesions, unravelling passive or adaptive changes upon immune-modulating regimens, and deploying single-cell analysis in prospective trials and clinical practice. The renewed insights are likely to offer novel opportunities for developing companion biomarkers to assign UBC patients into the most effective treatment modalities, and designing rational single or combination immunotherapies with improved response rate and prolonged overall survival.

## Author Contributions

JZ and KY contributed equally to the literature search, figure visualization, and manuscript drafting. YF and RZ helped with data curation. HC and GZ conceptualized and supervised the project. All authors contributed to the article and approved the submitted version.

## Funding

This work was supported by the National Natural Science Foundation of China (81672514, 81902562), Shanghai Natural Science Foundation (16ZR1420300, 18410720400, 19431907400), Ren Ji Hospital Research Funding Projects (RJZZ18-020, PYIII-17-017, PY2018-IIC-02), Shanghai Jiao Tong University School of Medicine Research Funding Projects (TM201708), and Foundation of Shanghai Hospital Development Center (SHDC12015125).

## Conflict of Interest

The authors declare that the research was conducted in the absence of any commercial or financial relationships that could be construed as a potential conflict of interest.
